# The Power of Far-Red Light at Night: Photomorphogenic, Physiological, and Yield Response in Pepper During Dynamic 24 Hour Lighting

**DOI:** 10.3389/fpls.2022.857616

**Published:** 2022-04-26

**Authors:** Jason Lanoue, Celeste Little, Xiuming Hao

**Affiliations:** Harrow Research and Development Centre, Agriculture and Agri-Food Canada, Harrow, ON, Canada

**Keywords:** dynamic 24h lighting, greenhouse pepper, far-red light, continuous light, light spectrum, light intensity, photomorphogenesis, photoinjury

## Abstract

Supplemental light is needed during the winter months in high latitude regions to achieve the desired daily light integral (DLI) (photoperiod × intensity) for greenhouse pepper (*Capsicum annuum*) production. Peppers tend to have short internodes causing fruit stacking and higher labor time for plant maintenance when grown under supplemental light. Far-red light can increase internode length, and our previous study on tomatoes (*Solanum lycopersicum*) also discovered monochromatic blue light at night during continuous lighting (CL, 24 h) increased stem elongation. Furthermore, the use of low-intensity, long photoperiod lighting can reduce light fixture costs and overall electricity costs due to lower power prices during the night. Therefore, we investigated the use of blue and/or far-red light during the night period of CL to increase stem elongation. Three pepper cultivars with different internode lengths/growing characteristics (‘Maureno,’ ‘Gina,’ and ‘Eurix’) were used to investigate the effects on plant morphology in a short experiment, and one cultivar ‘Maureno’ was used in a long experiment to assess the impact on fruit yield. The five lighting treatments that were used are as follows: 16 h of white light during the day followed by either 8 h of darkness (16W – control), white light (24W), blue light only (16W + 8B), blue + far-red light (16W + 8BFR), or far-red light only (16W + 8FR). Calculated nighttime phytochrome photostationary state (PSS) was 0.833, 0.566, 0.315, and 0.186 for 24W, 16W + 8B, 16W + 8BFR, and 16W + 8FR respectively. All five treatments had the same DLI in photosynthetically active radiation (PAR) and far-red light. The 16W + 8BFR and 16W + 8FR treatments significantly increased internode length compared to 16W and 24W but neither was more impactful than the other. The 16W + 8B treatment also increased internode length but to a lesser extent than 16W + 8BFR and 16W + 8FR. This indicates that a nighttime PSS of 0.315 is sufficient to maximize stem elongation. Both 16W + 8B and 16W + 8BFR drove photosynthesis during the nighttime supporting a similar yield compared to 16W. Therefore, 16W + 8BFR is the most potential lighting strategy as it can lead to a greater reduction in the light fixture and electrical costs while maintaining yield and enhancing internode length.

## Introduction

The daily light integral (DLI; light intensity × photoperiod duration) plays a vital role in plant biomass accumulation and yield. While the natural solar DLI is dictated by the time of year, global location, and local weather, the total DLI can be augmented by the application of supplemental lighting. Supplemental lighting can aid in the achievement of a desired/target DLI to increase plant growth and yield, specifically during low-light months (McAvoy et al., 1989). The timely use of lighting during low-cost periods can maximize economical gain for growers (Sørensen et al., 2020). Extended photoperiods (up to 24 h) with supplemental light at a lower light intensity reduce the overall fixture need (i.e., capital cost) and move part of daytime electricity use (or demand) to the nighttime when electricity prices are at their lowest; such is the case in Ontario, Canada (Hao et al., 2018a; IESO, 2022). Furthermore, regardless of fixture type [whether it is high-pressure sodium (HPS) or light-emitting diode (LED)] most of the input electricity in light fixtures is eventually converted into heat because plants only convert a small percentage of light energy into biomass (Kozai and Niu, 2016^1^). The law of conservation of energy states that energy can neither be created nor destroyed – only converted from one form of energy to another – i.e., a system always has the same amount of energy^2^ unless there is an exchange with outside. During the night when lighting is not used, heating is usually needed to maintain the proper greenhouse temperatures to prevent any low-temperature damage to plants. By utilizing LED lighting during the subjective night period, the heat generated from the application of lighting can help to meet nighttime heating requirements, reducing the use of fossil fuels for heating and associated carbon dioxide (CO_2)_ emission (Velez-Ramirez et al., 2012) during the night period. However, exceeding the tolerable limits of photoperiods, which are species-specific, can lead to diminished yield, photoperiod-related leaf injury, and an economic disadvantage for growers (Demers and Gosselin, 2002; Hao et al., 2018a). While lettuce (Ohtake et al., 2018) and cucumber (Lanoue et al., 2021b) are more tolerant to extended photoperiods including 24 h continuous lighting (CL), others such as tomato (Velez-Ramirez et al., 2014; Matsuda et al., 2016) and pepper (Demers and Gosselin, 2002) are susceptible to photoperiod related injury. Photoperiod injury can manifest in many ways including interveinal chlorosis which leads to lower photosynthetic rates and ultimately reductions in yield (Sysoeva et al., 2010; Velez-Ramirez et al., 2011). Unlike tomatoes which develop severe leaf chlorosis, pepper leaves tend to be less affected by CL, developing only mild or no chlorosis and subtle leaf deformities (Demers et al., 1998). However, a reduction in pepper yield is still observed under CL indicating that the plant is unable to utilize the additional photons (Demers et al., 1998). While the exact mechanism controlling photoperiod injury is unknown, research in tomatoes suggests that CL tolerance is conferred by a single gene; *type III light-harvesting chlorophyll a/b binding protein 13* (*CAB-13*; Velez-Ramirez et al., 2014). However, it should be noted that there are many circadian clock components that form complex interactions indicating that there are potentially many points of the regulation (Inoue et al., 2018). The potential mismatch between internal diurnal patterns of gene expressions and the lack of external light/dark cycles could also play a role in CL injury.

**FIGURE 1:**
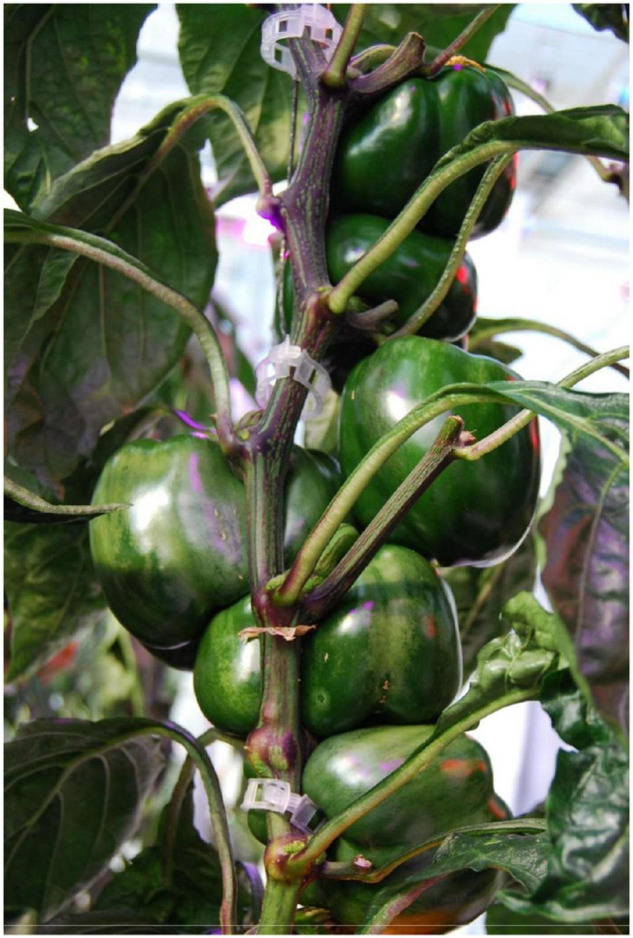
Fruit stacking due to short internodes in plants grown under supplemental lighting.

The increase in light intensity due to supplemental lighting during greenhouse production is known to cause plants to become more compact as additional light represses stem elongation and internode length (Hao and Papadopoulos, 1999; Fan et al., 2013). This plant compaction is further observed in peppers when they are grown under CL (Demers et al., 1998). For peppers, this increased plant compaction, specifically a reduction in internode length, may cause fruit stacking which can negatively affect fruit shape (Figure 1) and lead to increased labor cost due to the difficulty in removing suckers during plant maintenance. Short internode has been identified as a limiting factor for winter cultivation (such as from December to February) with bell peppers under supplemental light.

Several studies have reported the usefulness of wavelength-specific LEDs, particularly the use of far-red (FR) light, to promote stem elongation and leaf expansion in many species including peppers (Brown et al., 1995; Rajapakse and Li, 2004; Kalaitzoglou et al., 2019). By increasing the amount of FR light, the phytochrome photostationary state (PSS) defined as the ratio of phytochrome in the active state (P_*fr*_) to the total phytochrome in the active and inactive state (P_*fr*_ + P_*r*_; Sager et al., 1988) under a given light spectra, can be altered. A low PSS will invoke a shade avoidance response leading to increased stem elongation (Cole et al., 2011).

Alterations in the PSS can lead to changes in biomass partitioning. Similar to the shade avoidance response to stem elongation, the addition of FR light can also result in an increase in biomass partitioning of the stem (Brown et al., 1995; Maliakal et al., 1999; Ji et al., 2019, 2020). Furthermore, the addition of FR light has been shown to increase dry mass partitioning to fruit, thus increasing yield, which in vascular plants can indicate preferential carbon export to the fruit (Lanoue et al., 2018a; Kalaitzoglou et al., 2019; Ji et al., 2020; Jin et al., 2021). It should be noted that while phytochrome is traditionally thought of as a photoreceptor for red and FR, it does absorb other wavelengths which can affect the PSS (Eilfeld and Rüdiger, 1985; Sager et al., 1988; Chun et al., 2001; Sullivan et al., 2016).

The use of short-term (typically 15–60 min) end-of-day FR (EOD-FR) light has also been proposed as an alternative to the use of FR during the day (Kasperbauer et al., 1984; Chia and Kubota, 2010; Islam et al., 2014; Kalaitzoglou et al., 2019). EOD-FR treatments provide low PSS as FR is used during a period absent of photosynthetically active radiation (PAR). An EOD-FR treatment typically uses a low FR light intensity which is viewed as more energy efficient in comparison to daytime FR application, while also can invoke a shade avoidance response (i.e., stem elongation and leaf expansion) (Chia and Kubota, 2010; Kalaitzoglou et al., 2019). However, while EOD-FR has been shown to increase light use efficiency (i.e., dry matter accumulated per cumulative incident light; Zou et al., 2019), its use does not have the same impact on plant height as moderate-intensity (∼50 μmol m^–2^ s^–1^) FR light when used during the day. Kalaitzoglou et al. (2019) showed that an EOD-FR treatment of 17 μmol m^–2^ s^–1^ increased plant height compared to a control treatment without FR; however, the plants are more compact when compared to the use of continuous daytime FR at 54 μmol m^–2^ s^–1^. Zou et al. (2019) also showed that in lettuce, the use of 50 μmol m^–2^ s^–1^ of FR during a 16 h photoperiod produced larger plants than the use of a 1-h 50 μmol m^–2^ s^–1^ EOD-FR treatment. In peppers that may have internode lengths as low as 2.9 cm when grown under supplemental lighting (Demers et al., 1998), a subtle increase in stem elongation due to EOD-FR would not be sufficient to increase internode length to prevent fruit stacking. Therefore, we hypothesize that the use of low intensity FR light for a longer duration during the night in a dynamic CL strategy (i.e., changes in light intensity and/or spectrum between day and night periods) would provide the internode stretch needed to avert fruit stacking and reduce additional labor cost for plant maintenance.

Blue light (400–500 nm) is traditionally associated with reduced plant height when added to a lighting treatment (Shimizu et al., 2006; Nanya et al., 2012; Hernández and Kubota, 2016). However, a unique phenomenon occurs when blue light is used as a sole source. In the absence of other wavelengths, monochromatic blue light has been shown to increase stem elongation in cucumber (Hernández and Kubota, 2016), tomato (Lanoue et al., 2019), and *Arabidopsis* (Kong and Zheng, 2020). This phenomenon is presumably the opposite of what one would traditionally expect. In the absence of other light, monochromatic blue light can have a low PSS which is known to cause stem elongation (Cole et al., 2011; Kalaitzoglou et al., 2019; Kong and Zheng, 2020). Furthermore, Kong and Zheng (2020) suggest that phototropin II is involved in stem elongation as the absence of this photoreceptor in *Arabidopsis* produced taller plants than the wild-type when grown under blue light. Therefore, sole irradiation with blue light (such would be the case during the night) can increase stem elongation (Hernández and Kubota, 2016; Lanoue et al., 2019; Kong and Zheng, 2020).

The use of dynamic CL lighting has shown promise in other crops in averting photoperiod related injury (Matsuda et al., 2016; Ohtake et al., 2018) and allowed injury-free production in greenhouse tomatoes (Lanoue et al., 2019, 2021a) but no research has been done on greenhouse peppers yet. Therefore, this study was conducted to investigate the effects of dynamic CL lighting with monochromatic blue, far-red or blue + far-red light during the night on plant growth, morphology, leaf gas exchanges, and fruit yield of pepper plants to identify dynamic CL or 24 h lighting strategies that can reduce leaf injury, increase internode length/stem elongation and maintain high fruit yield and quality. The lighting strategies can generate significant economic benefits if the high yield and quality under lighting can be maintained. This is because low-intensity CL reduces light fixture capital cost due to the lower intensity of light utilized to provide the same DLI as the conventional shorter photoperiods of lighting, the lower electricity price during the off-peak period at night, and the lower peak power demand charge (Hao et al., 2018a; IESO, 2022).

## Materials and Methods

### Plant Material and Experimental Design

Two experiments were conducted in this study. The first experiment was aimed at identifying dynamic CL strategies which could prevent injury while either maintaining or increasing fruit yield and quality. Only one cultivar was used in this experiment because of the large space (large plants) requirement of the experiment. The second experiment expanded to three cultivars with different growth characteristics and investigated the effects of different spectra of light during the night on plant morphology/architecture (especially on internode length) and biomass partitioning in young pepper plants. Taken together, the two experiments provided more complete information on the impact of dynamic CL with different spectra of light during the night on the photomorphogenesis, physiology, and yield of pepper plants.

#### Experiment One

Forty-one-day-old pepper (*Capsicum annuum*) transplants cv. ‘Maureno’ were planted onto rockwool slabs on October 5th, 2020 in two adjacent double-layer polyethylene greenhouses (50 m^2^ of growing area each) at the Harrow Research and Development Centre (Agriculture and Agri-Food Canada, Harrow, ON, Canada; 42.03°N, 82.9°W). Plants were trained in a high wire double-stem fashion at a plant density of 7.05 stems m^–2^. In total, 72 plants were placed under each of the four light treatments. The plants were drip-irrigated as needed using a complete nutrient solution (Ontario Ministry of Agriculture, Food and Rural Affairs [OMAFRA], 2010) with an electrical conductivity of 2.8 dS m^–1^ and a pH of 5.9. The greenhouse was enriched to 800 μL L^–1^ of CO_2_ during both day and night when the greenhouse was not vented (actual concentrations 800–1000 μL L^–1^). The heating temperature during the day was 20.5°C with a venting temperature of 25°C (actual temperature 20.5–25°C, Supplementary Figure 1A). Day humidification set point was 65% with a dehumidification set point of 85% (actual humidity 62–80%, Supplementary Figure 1B). The nighttime heating temperature was 18°C and venting was 22°C (actual temperature 18.5–20.5°C). Night humidification set point was 55% with a dehumidification set point of 80% (actual humidity 55–65%).

**FIGURE 2:**
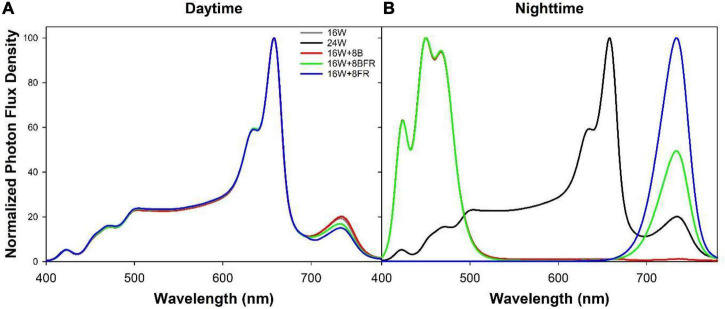
Normalized photon flux density (PFD) of daytime **(A)** and nighttime **(B)** light treatments as determined with a Li-180 spectroradiometer during the night at the head of the plant.

Each greenhouse was subdivided into four sections by light abatement curtains (Obscura 9950 FR W, Ludvig Svensson, Kinna, Sweden). The light abatement curtains were closed on cloudy days and during the night to prevent treatment contamination. On sunny days, the light abatement curtains were opened to prevent the shading of the high-intensity solar radiation. Guard plants were utilized on the outermost rows of each greenhouse. Beginning on November 12th, 2020, four overhead supplemental light treatments (Table 1) were implemented with Sollum SF04 LED lighting fixtures (Sollum Technologies Inc., Montréal, QC, Canada). These fixtures have multiple independently controlled diode channels which allow for changes in spectrum and intensity throughout a 24 h period. The light treatments were as follows: 16 h of white light (3257k) during the day followed by 8 h of darkness (control; 16W), continuous white light for 24 h which represents the largest possible reduction in fixture cost (24W), 16 h white light during the day followed by 8 h of blue light (peak wavelength = 451 nm) at night which allowed us to test the hypothesis that sole blue light can increase stem elongation (16W + 8B), and 16 h of white light during the day followed by 8 h of blue and far-red light (peak wavelength = 734 nm) during the night to observe if there was an additive effect of the two wavelengths on stem elongation (16W + 8BFR; Figure 2). All daytime white treatments provided the same percentage of blue (400–499 nm), green (500–599 nm), and red (600–699 nm; 12.1% blue, 30.2% green, and 57.7% red) but at different total intensities (Table 1). Daytime was defined as 6:00–22:00 and nighttime was defined as 22:00–6:00. The light in each treatment was measured at four locations within each plot in each greenhouse with a 1-m quantum light sensor (Li-COR 191R; Li-COR Biosciences, Lincoln, NE, United States) just above the apex of the plant. The light intensity was adjusted *via* automatic dimming of the light fixtures as needed throughout the experiment to maintain the appropriate light levels (Table 1). Spectral composition readings were taken at the apex of the plant using a Li-COR Li-180 spectroradiometer (Figure 2). The daily light integrals (DLI) for photosynthetically active radiation (PAR, 12.6 ± 0.1 mol m^–2^ d^–1^) and for far-red light (FR, 1.41 ± 0.06 mol m^–2^ d^–1^) were the same for all four lighting treatments (Table 1). Supplemental lighting accounted for 47–81% of total DLI depending on solar radiation fluctuations throughout the growing period. The far-red light during the daytime in 16W + 8BFR was reduced accordingly so that same DLI for far-red light could be achieved. PSS values were calculated following the methods of Sager et al. (1988).

**TABLE 1:** Supplemental light intensities provided by Sollum SF04 fixtures of lighting treatments in experiments one and two where the daytime is defined as 6:00-22:00 and nighttime is 22:00-6:00.

Treatment	16W	24W	16W + 8B	16W + 8BFR	16W + 8FR
**Experiment 1**					
Daytime PAR (μmol m^–2^ s^–1^)	220 ± 4	147 ± 3	181 ± 4	180 ± 5	–
Daytime FR (μmol m^–2^ s^–1^)	24 ± 3	17 ± 2	22 ± 2	18 ± 2	–
Daytime PSS	0.834	0.833	0.832	0.840	–
Nighttime PAR (μmol m^–2^ s^–1^)	–	147 ± 3	73 ± 2	74 ± 3	–
Nighttime FR (μmol m^–2^ s^–1^)	–	17 ± 2	–	16 ± 3	–
Nighttime PSS	–	0.833	0.566	0.315	–
PAR DLI (mol m^–2^ d^–1^)	12.7 ± 0.2	12.7 ± 0.3	12.5 ± 0.3	12.5 ± 0.4	–
FR DLI (mol m^–2^ d^–1^)	1.38 ± 0.17	1.47 ± 0.17	1.27 ± 0.12	1.50 ± 0.21	–
**Experiment 2**					
Daytime PAR (μmol m^–2^ s^–1^)	180 ± 6	121 ± 3	153 ± 4	152 ± 5	182 ± 5
Daytime FR (μmol m^–2^ s^–1^)	19 ± 2	13 ± 1	18 ± 2	13 ± 2	13 ± 1
Daytime PSS	0.834	0.833	0.832	0.840	0.844
Nighttime PAR (μmol m^–2^ s^–1^)	–	121 ± 3	55 ± 2	56 ± 2	–
Nighttime FR (μmol m^–2^ s^–1^)	–	13 ± 1	–	11 ± 1	10 ± 1
Nighttime PSS	–	0.833	0.566	0.315	0.186
PAR DLI (mol m^–2^ d^–1^)	10.4 ± 0.3	10.4 ± 0.3	10.4 ± 0.3	10.4 ± 0.3	10.5 ± 0.3
FR DLI (mol m^–2^ d^–1^)	1.14 ± 0.12	1.14 ± 0.09	1.04 ± 0.12	1.07 ± 0.14	1.04 ± 0.09

*Dashes (–) represent no PAR or FR light during that period. PSS was calculated using Sager et al. (1988) via data obtained from a Li-180 spectroradiometer during the night at the apex of the plant in order to eliminate confounding effects of the sunlight.*

All light measurements were performed at night to avoid any contamination from solar radiation. Lights remained on regardless of the natural solar radiation levels to ensure the same DLIs for all lighting treatments. Plants from experiment one were used for all gas exchange measurements, chlorophyll fluorescence measurements, and fruit production evaluation. The experiment was terminated at the end of March because our past research indicates that the effect of supplemental far-red light on greenhouse peppers is negligible after mid-March when there is high intensity of far-red light from sunlight (Hao et al., 2018a).

#### Experiment Two

Pepper seeds cv. ‘Maureno,’ ‘Gina,’ and ‘Eurix’ were sown into 1 cm × 1 cm rockwool propagation cubes on March 29th, 2021 and placed in a germination chamber with 100% humidity for 8 days. Upon germination, seedlings were transplanted into 2-l pots filled with soil media (BM6, Berger, Saint-Modeste, QC, Canada) and placed in a greenhouse. On April 26th, 2021 40 plants of each cultivar were placed under one of five light treatments (120 plants encompassing all three cultivars under each treatment) in two double-layer polyethylene greenhouses. A fifth treatment was added to better understand the role of sole FR light during the night and its impact on stem elongation (16W + 8FR). The 16W + 8FR treatment represented 16 h of white light followed by 8 h of sole far-red light (Figure 2 and Table 1). The greenhouse roof was covered with light abatement curtains (Obscura 9950 FR W, Ludvig Svensson, Kinna, Sweden) which blocked 90–95% of incoming solar radiation. Measured solar light intensity during solar noon was between 20 and 30 μmol m^–2^ s^–1^ inside the greenhouse. In this way, we could assess the biomass partitioning and growth patterns of peppers without interference from the sun (i.e., in a growth chamber-like setting). Lighting was again provided by Sollum SF04 fixtures. All five lighting treatments had the same PAR DLI (10.4 ± 0.01 mol m^–2^ d^–1^) and FR DLI (1.09 ± 0.03 mol m^–2^ d^–1^) measured just above the apex of the plants (Figure 2 and Table 1). Supplemental lighting accounted for between 89 and 96% of the total DLI the plant was exposed to, depending on daily solar radiation. PSS values were calculated following the methods of Sager et al. (1988). The 16W, 24W, 16W + 8B, and 16W + 8BFR treatments were similar to experiment one but had a lower overall DLI. The irrigation management and greenhouse climate parameters were similar to experiment one (Supplementary Figure 2). Plants in experiment two were used for plant morphological and biomass partitioning assessment.

### Leaf Gas Exchange: Day and Night Measurements

As stated above, all gas exchange data were obtained using plants from experiment 1. Thus the days into the treatment (DIT) are calculated from November 12th, 2020. For day and nighttime measurements, the fifth leaf was placed in the chamber of a Li-COR 6400 (Li-COR Inc., Lincoln, NE, United States) which was fitted with a 2-cm by 3-cm clear top chamber. The leaf temperature was set to 22°C with a relative humidity of 60–70% and a CO_2_ level held at 800 μL L^–1^. Four leaves from separate plants under each treatment were used at 99 DIT for both day and nighttime measurements. Daytime measurements were performed on cloudy days to maximize the effect of supplemental lighting while minimizing the effect of natural sunlight. Nighttime measurements began half an hour after lighting changes (22:30), allowing the leaves to acclimate to the new light environment. Leaves were kept in the chamber until a steady-state photosynthesis rate was obtained, then the average from 2 min was taken.

### Leaf Gas Exchange: Light Response Curves

The fifth leaf was placed in the chamber of a Li-COR 6400 photosynthesis system which was fitted with a 2-cm by 3-cm red/blue (88%R/12%B) LED Li-COR standard light source. The leaf temperature was set to 22°C with a relative humidity of 60–70% and a CO_2_ level held at 800 μL L^–1^. Four leaves from separate plants under each treatment were used at 91 DIT. Measurements were performed on cloudy days. Leaves were acclimated to high light intensity (1500 μmol m^–2^ s^–1^) until a steady-state photosynthetic rate was achieved. After the steady-state was achieved, light curves began at a high light intensity and decreased gradually following the procedure from Lanoue et al. (2018b). At each light level, the photosynthetic rate was allowed to reach a steady-state, then measurement was taken for that light level. Photosynthetic rates were plotted against the light intensity and fitted to a regression line following the equation y = y_*o*_ + a(1-e^(–b^*x)^) using SigmaPlot 10.0 to determine the photosynthetic maximum. A linear regression (y = mx + b) using the photosynthetic rates between the light levels of 0–100 μmol m^–2^ s^–1^ was used to calculate both the light compensation point (LCP) and quantum yield (QY).

### Chlorophyll Fluorescence Imaging

Intact leaves were dark-adapted using aluminum foil for 20 min. After the dark adaptation period, leaflets were detached and immediately used for chlorophyll imaging using a closed FluorCam model FC 800-C with FluorCam v.7.0 software (FluorCam, Photon System Instruments, Brno, Czechia). The minimum fluorescence in a dark-adapted state (F_*o*_) was acquired during a dark period of 5 s, after that, an 800 ms saturating light pulse (3000 μmol m^–2^ s^–1^) from a blue LED (peak emission of 449 nm) was used to measure the maximum fluorescence in a dark-adapted state (F_*m*_). From F_*o*_ and F_*m*_, the variable fluorescence in a dark-adapted state (F_*v*_) was calculated (F_*v*_ = F_*m*_-F_*o*_) which was used to determine the maximum photosystem II (PSII) quantum yield (F_*v*_/F_*m*_). In general, the lower the value of F_*v*_/F_*m*_, the more severe the photo-inhibition and thus, the leaf injury (Baker, 2008). By calculating F_*v*_/F_*m*_ using chlorophyll fluorescence imaging, we are able to assess not only the prevalence of injury but also the spatial heterogeneity of F_*v*_/F_*m*_ from a leaflet. Eight leaflets from the fifth leaf of different plants were used for each light treatment when plants were at 110 DIT.

### Yield

Yield analysis was performed on plants from experiment one only. Harvest was performed weekly throughout the experiment beginning on December 31st, 2020 (50 DIT) and finishing on March 24th, 2021 (133 DIT) with peppers being harvested once they reached full size and had gone through a 75% color change.

### Biomass Partitioning and Destructive Measurements

Biomass partitioning and destructive harvest measurements were performed on plants from experiment two only. Measurements were done from June 1–3, 2021 (37–39 DIT) and again from June 28–30, 2021 (64–66 DIT) with ten and six plants respectively from each cultivar. During these measurements, plant height, internode length, leaf number, leaf area, and SPAD values of the fifth leaf were determined. Plant height was measured as the distance from the base of the plant in the soil to the top of the tallest stem on that plant. Internode length was the distance from the highest node which contained a leaf longer than 5 cm on the tallest stem to the bifurcation point (i.e., where two stems naturally split). The distance was then divided by the number of nodes between these two points to determine internode length. The leaf area was determined using a Li-COR 3100 (Li-COR Inc.) leaf area meter. SPAD value was determined using a chlorophyll meter taking six measurements on each leaf to determine an average for that leaf (SPAD model 502, Konica Minolta, Osaka, Japan). The leaves and stems were separated from the plant and weighed (fresh weight), then placed in an oven at 65°C for 2 weeks before being weighed for dry weight.

### Statistical Analysis

All statistics were performed using SAS Studio 3.5. After the ANOVA, multiple means comparisons between the different treatments were done using a Tukey-Kramer adjustment and a value of *p* < 0.05 to indicate a significant difference. Regression analysis was done using a backward elimination method in SAS Studio 3.5. Final regressions with a *p* < 0.05 were determined to be significant.

## Results

### Leaf Gas Exchange and Chlorophyll Fluorescence

**FIGURE 3:**
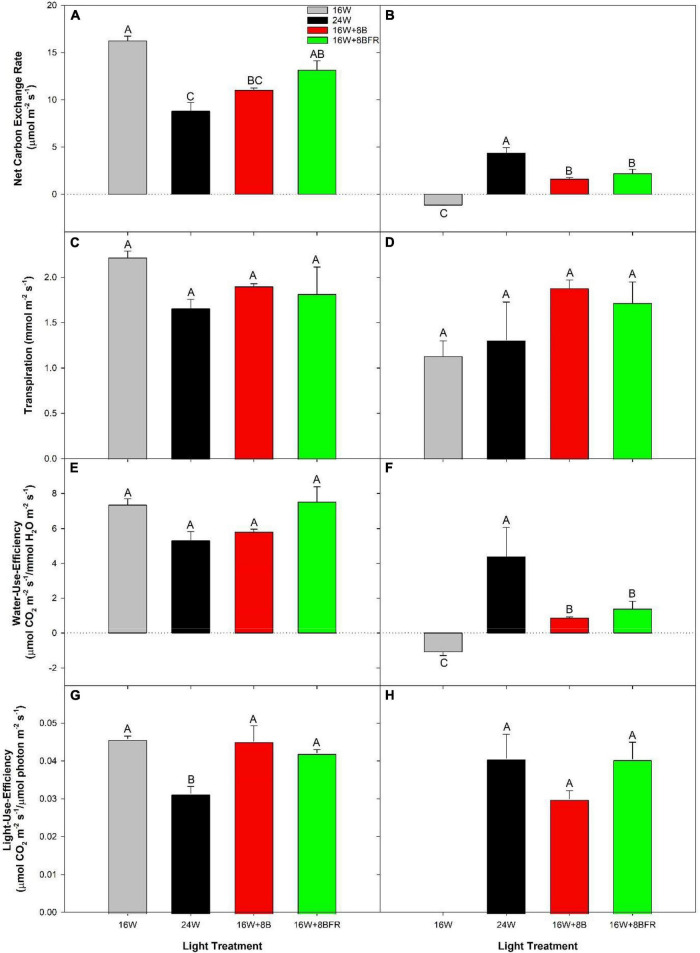
Net carbon exchange rates **(A,B)**, transpiration rates **(C,D)**, water-use-efficiency (WUE; **E,F**), and light-use-efficiency (LUE; **G,H**) of the fifth leaf from pepper plants grown under the lighting treatments at 99 DIT during the daytime **(A,C,E,G)** and nighttime **(B,D,F,H)**. Daytime measurements were performed using a Li-COR 6400 fitted with a clear top chamber on a cloudy day and thus represent the photosynthesis driven mostly by the supplemental lighting. Nighttime measurements were performed after 22:30 (half an hour after night lighting started), allowing leaves to adjust to the new light environmental conditions. Error bars represent the standard errors of the means of *n* = 4. Letter groups (A, B, C) represent a significant difference within a panel between the lighting treatments (*p* < 0.05).

Clear top photosynthetic measurements were done on cloudy days which allowed for the observation of how each light treatment affected light capture and gas exchange directly (Figure 3). Leaves grown under 16W + 8BFR produced similar photosynthetic rates to the control, 16W (Figure 3A). Leaves from both 24W and 16W + 8B produced lower photosynthetic rates than the control (16W). There was no significant difference among all treatments on leaf transpiration rates (Figure 3C) and the ratios of CO_2_ influx vs. H_2_O efflux (water-use-efficiency; WUE) (Figure 3E). Calculation of light-use-efficiency (LUE) was also performed which normalized photosynthesis on an incoming photon basis. In this way, we can compare photosynthetic capacity between the light treatments with different intensities. This parameter also allows us to remove any small effects of changing solar radiation intensity, even though these measurements are taken on cloudy days. The 16W, 16W + 8B, and 16W + 8BFR treatments had similar LUE while 24W was lower (Figure 3G). This result indicated that 24W was less efficient at turning incident light into assimilated carbon. Also, even though 16W + 8B was observed to have a lower photosynthetic rate than 16W, it was mainly due to a lower light intensity of the treatment during daytime (Table 1).

Leaves from 16W produced a negative net carbon exchange rate (NCER) representing respiration during the night period (Figure 3B), as expected. However, leaves from 24W, 16W + 8B, and 16W + 8BFR all produced positive NCERs, indicating photosynthesis. These photosynthetic rates during the night period coupled with daytime photosynthetic rates indicate positive carbon assimilation for a continuous 24 h period. It should be noted that 24W had the highest NCER of all treatments during the night which corresponded with the highest light intensity during that period (Figure 3B). Transpiration rates during the night period were similar to all light treatments (Figure 3D). As the quotient of NCER and transpiration, WUE closely follows the patterns of NCER with 16W producing the lowest WUE and 24W the highest (Figure 3F). LUE values for 16W were non-resultant because there was no light during the nighttime (Figure 3H). The LUEs for the three treatments with lighting during the night (24W, 16W + 8B, and 16W + 8BFR) were similar (Figure 3H), indicating the photosynthetic capacity of the three treatments was the same and the difference in their net carbon exchange rates was due to difference in night light intensity (Table 1).

**FIGURE 4:**
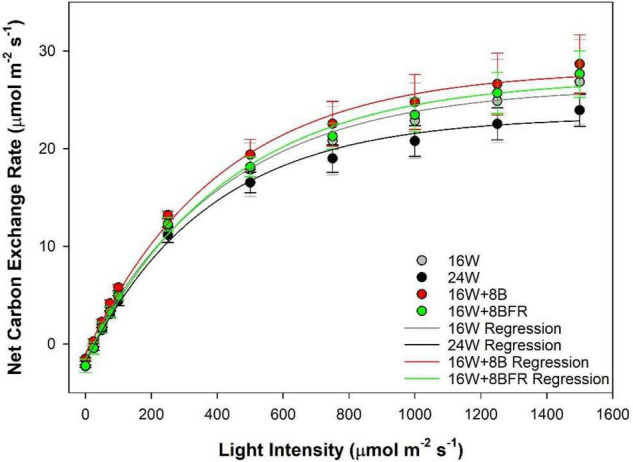
Leaf photosynthetic light response curves of pepper plants grown under the light treatments at 91 DIT as determined using a Li-COR 6400 photosynthesis system with a red/blue standard Li-COR light source. Measurements were made at a CO_2_ level of 800 μL L^–1^, leaf temperature of 22°C, and relative humidity of 60–70%. Regressions lines were fit to y = y_*o*_ + a(1-e^(–b^*x)^) for each lighting treatment.

Photosynthetic light response curves (Figure 4) allow for the assessment of how plants grown under different light treatments respond to the different light intensities. The light compensation point (LCP) is the light intensity at which the photosynthetic rate and the respiratory rate are equal to each other (i.e., no net CO_2_ gain or loss). Under all treatments, the LCP was similar indicating that plants are able to start carbon assimilation at roughly the same light intensity (Table 2). Similar to LUE, apparent quantum yield is a metric that determines how much CO_2_ is assimilated with each additional photon added. This parameter is calculated during the linear phase (0–100 μmol m^–2^s^–1^) of light increase. Continuous lighting had no detrimental effect on apparent quantum yield (Table 2). The photosynthetic maximum (Pn_*max*_) is a measurement of the maximum photosynthetic rate when light is not a limiting factor. In this sense, it is a proxy for what the plant may encounter during periods of intense natural solar radiation. Similar to other metrics of the light response curve, Pn_*max*_ was comparable among all treatments, indicating that the photosynthetic performance under strong light was not affected by continuous illumination (Table 2).

**TABLE 2:** Summary of the major physiological parameters as determined by leaf light response curves (Figure 4) from peppers grown under all treatments at 91 DIT.

Treatment	Light compensation point (μmol m^–2^ s^–1^)	Apparent quantum yield (μmol CO_2_ m^–2^ s^–1^/μmol m^–2^ s^–1^)	Pn_*max*_ (μmol CO_2_ m^–2^ s^–1^)
16W	21.42 ± 3.78^A^	0.069 ± 0.006^A^	27.34 ± 4.65^A^
24W	32.19 ± 3.30^A^	0.068 ± 0.004^A^	25.14 ± 1.81^A^
16W + 8B	21.04 ± 3.25^A^	0.075 ± 0.001^A^	29.38 ± 3.90^A^
16W + 8BFR	29.22 ± 8.14^A^	0.072 ± 0.004^A^	29.17 ± 2.94^A^

The light compensation point (LCP) and apparent quantum yield (QY) were calculated from a regression line (y = mx + b) fitted to the values between the PAR values of 0–100 μmol m–^2^ s–^1^. The photosynthetic maximum (Pn_max_) was calculated from y = y_o_ + a(1-e^(–b^*x)^). Values ± the standard errors of the means are representative of n = 4. Within each parameter and measurement date, different letters represent a statistical difference as determined by a one-way ANOVA with a Tukey-Kramer adjustment (p < 0.05).

**FIGURE 5:**
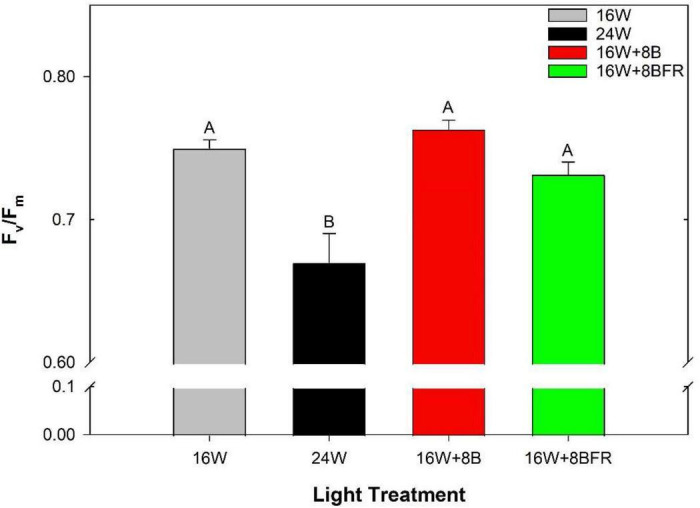
Maximum efficiency of PSII (F_*v*_/F_*m*_) of the fifth leaf from peppers grown under all light treatments at 110 DIT. Error bars represent the standard errors of the means of *n* = 8. Letter groups (A, B) represent a statistical difference as determined by a one-way ANOVA with a Tukey-Kramer adjustment (*p* < 0.05).

**FIGURE 6:**
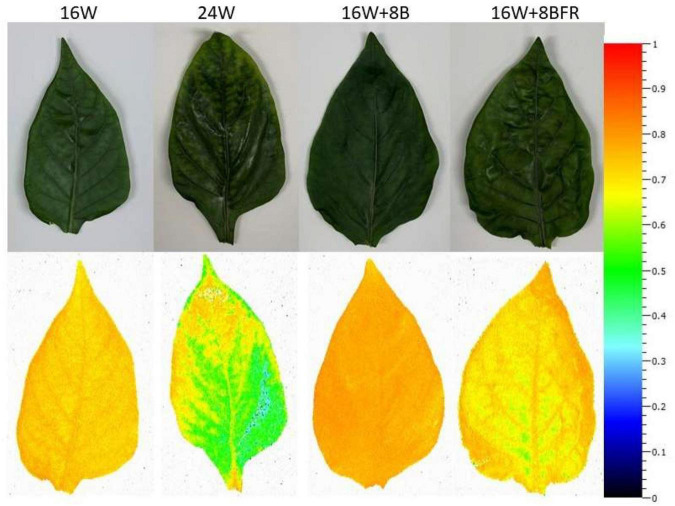
Spatial response of F_*v*_/F_*m*_ from the fifth leaf of peppers grown under all light treatments measured at 110 DIT.

As stated previously, chlorophyll fluorescence measurements are an unbiased determination of the stress status of a leaf in comparison to visual chlorosis ratings. The maximum quantum efficiency of PSII photochemistry (F_*v*_/F_*m*_) is a parameter that is often used to monitor the stress of a leaf through the measurement of photo-inhibition. The higher the value, the less stressed a leaf is (Demmig and Björkman, 1987; Baker, 2008). Leaves grown under 24W have significantly lower F_*v*_/F_*m*_ values than all other treatments, indicating a higher level of stress, even if this was not visually apparent to the human eye (Figures 5, 6). Furthermore, fluorescence imaging allows for the assessment of the spatial impact of stress. In the bottom image of Figure 6, a large area of green is apparent in the image of the leaf grown under 24W, indicating a lower F_*v*_/F_*m*_ value and more stress. While 16W + 8BFR also displays some green coloration, it was much less severe. There was no significant difference in Fv/Fm values between 16W + 8BFR and W16 or 16W + 8B (Figure 4).

### Yield

**FIGURE 7:**
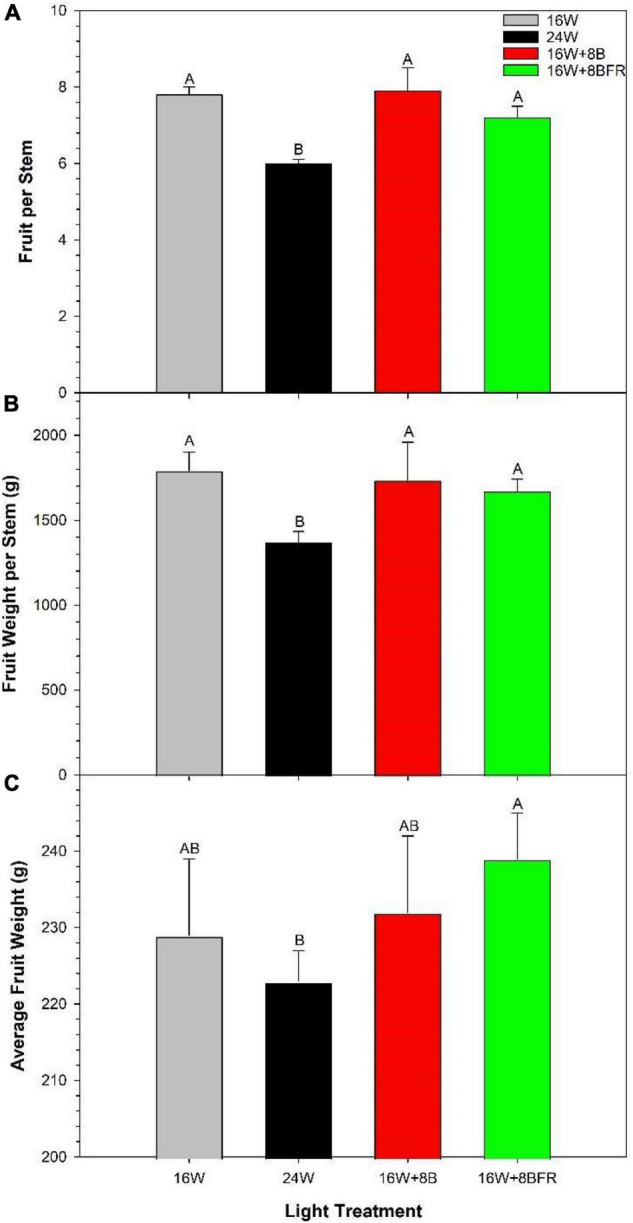
Total yield parameters from peppers grown under all light treatments from October 5th, 2020 to March 24th, 2021. **(A)** Represents the accumulated number of fruit harvested per stem, **(B)** represents the accumulated number of fruit harvested per stem, and **(C)** represents the average weight per fruit (fruit size). Within each panel, letter groups (A, B) represent a statistical difference as determined by a one-way ANOVA with a Tukey-Kramer adjustment (*p* < 0.05).

Ultimately, the feasibility of dynamic CL/24 h lighting hinges on the production of equal to or greater yield than a traditional 16h photoperiod (control, 16W). In Figures 7A,B, both 16W + 8B and 16W + 8BFR supported similar fruit production (total fruit number and weight per stem) to 16W (control) while 24W significantly reduced fruit production. The fruit size (average weight per fruit), a metric that often affects fruit grade was lower in 24W than in 16W + 8BFR (Figure 7C) while there was no difference among other treatments. Therefore, 16W + 8B and 16W + 8BFR were able to achieve the same fruit yield and grade as the control 16W while 24W reduced fruit yield and grade.

### Plant Morphology

**FIGURE 8:**
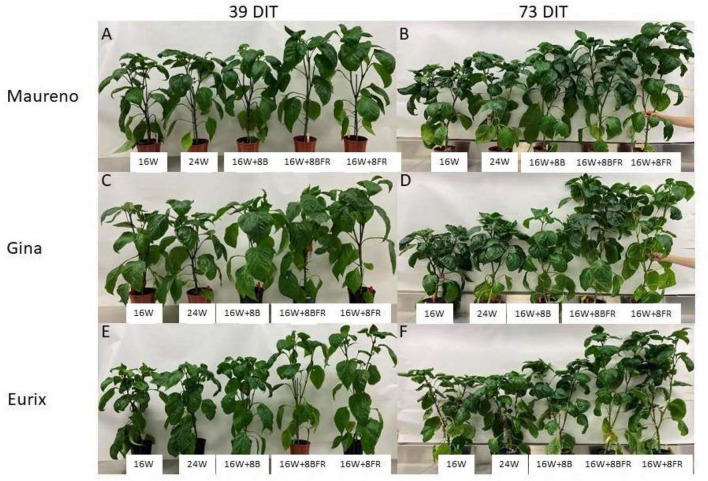
Pepper plants cv. ‘Maureno’ **(A,B)**, ‘Gina’ **(C,D)**, and ‘Eurix’ **(E,F)** grown under five different light treatments at 39 DIT **(A,C,E)** and 73 DIT **(B,D,F)** from experiment two.

**FIGURE 9:**
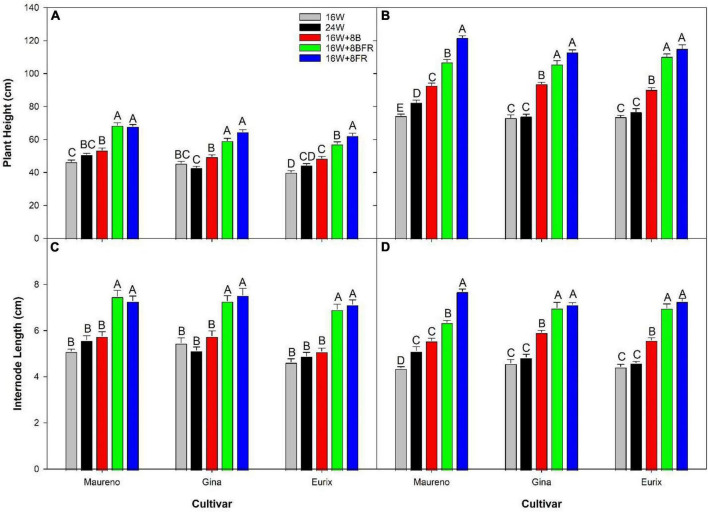
Plant height **(A,B)** and internode length **(C,D)** for cv. ‘Maureno,’ ‘Gina,’ and ‘Eurix’ from experiment two measured at either 37–39 DIT **(A,C)** or 64–66 DIT **(B,D)**. Values and standard errors in **(A,C)** represent ten plants while those in **(B,D)** represent six plants. Within each panel and cultivar, letter groups (A, B, C, D, E) represent a statistical difference as determined by a one-way ANOVA with a Tukey-Kramer adjustment (*p* < 0.05).

Experiment two was conducted to get an in-depth assessment of the morphological changes brought about by the various light treatments (Figure 8) using three cultivars with different growth architecture (especially internode length). ‘Maureno’ and ‘Gina’ in combination with 16W + 8FR and 16W + 8BFR produced the tallest plants while both 16W and 24W produced the shortest at 37-39 DIT (Figures 8A,C,E, 9A). Interestingly, in ‘Eurix’ 16W + 8FR produced taller plants than 16W + 8BFR. During the 64-66 DIT measurement period, differences in plant height due to light treatments were much more apparent. Again, both 16W + 8BFR and 16W + 8FR produced the tallest plants while 16W and 24W produced the shortest (Figures 8B,D,F, 9B). In fact, plant height increased by 63, 54, and 56% when comparing 16W + 8FR to 16W for ‘Maureno,’ ‘Gina,’ and ‘Eurix’ respectively. Furthermore, during both measurement periods, blue light at night (16W + 8B) increased stem height compared to 16W. Far-red light during the night either used as a sole source (16W + 8FR) or in addition to blue light (16W + 8BFR) resulted in a further increase (Figures 8, 9).

During the measurement period of 37–39 DIT, internode length was similar between 16W, 24W, and 16W + 8B in all cultivars (Figure 9C). Treatments 16W + 8BFR and 16W + 8FR produced similar internode lengths which were longer than those observed in plants grown under 16W, 24W, or 16W + 8B. Notably, 16W + 8BFR and 16W + 8FR both implemented FR light during the night either with blue light or as a sole source. At 64–66 DIT, 16W + 8FR again produced the longest internode for all three cultivars, and 16W + 8BFR produced similarly long internodes in ‘Gina’ and ‘Eurix’ (Figure 9D). Furthermore, plants are grown under 16W + 8B with monochromatic blue light at night also showed longer internode length when compared to 16W in all three cultivars (Figure 9D). This indicates that long-term exposure to blue light during the night period can increase internode length in pepper plants. The plants in 16W + 8BFR and 16W + 8FR had similar plant heights and internode lengths during both measurement periods (Figure 9) in all three cultivars except for a small difference in ‘Maureno,’ indicating the response of plant height and internode length between 16W + 8BFR and 16W + 8FR were mostly similar, and there was a minor influence of cultivars on the response.

Leaf area can give an indication of the light capture ability of a plant which can be impacted by the light environment. Between 37 and 39 DIT, the plants are grown under 24W and 16W + 8B produced the largest leaf area while the plants under 16W + 8FR had the lowest leaf area in ‘Gina’ (Table 3). Coincidentally, at 64–66 DIT all cultivars had the highest leaf area when grown under 16W + 8B which had sole blue light during the night treatment (Table 3). Both 16W + 8BFR and 16W + 8FR treatments that contained FR light were also observed to have an increased leaf area compared to 16W. At 37–39 DIT and in ‘Maureno’, the leaf number was similar among the treatments. In ‘Gina’ and ‘Eurix,’ 16W plants had the highest leaf number while 16W + 8BFR or both 16W + 8BFR and 16W + 8FR had the lowest numbers of leaves. At 64–66 DIT, 16W resulted in the highest number of leaves almost for all cultivars. In ‘Maureno,’ 16W + 8B also produced a high number of leaves, and in ‘Gina’ 24W produced a similar number of leaves as 16W (Table 3). For all three cultivars, treatments that contained far-red (solely or in combination with blue) produced the least number of leaves. Interestingly, at 64–66 DIT, 16W produced the most leaves in all cultivars but had the lowest leaf area indicating that the average leaf size was the smallest in the treatment.

**TABLE 3:** Leaf area, leaf number, specific leaf area, and SPAD value of ‘Maureno,’ ‘Gina,’ and ‘Eurix’ during the 37–39 DIT and 64–66 DIT measurement periods under different light treatments.

Cultivar	Treatment	Leaf Area (cm^2^)	Leaf number	Specific Leaf Area (m^2^ kg^–1^)	SPAD value
**37–39 DIT**					
Maureno	16W	2448 ± 106^A^	33.8 ± 1.7^A^	30.8 ± 0.9^B^	47.9 ± 1.1^A^
	24W	2513 ± 121^A^	32.4 ± 1.1^A^	40.0 ± 0.9^A^	46.4 ± 1.0^AB^
	16W + 8B	2648 ± 63^A^	32.9 ± 1.5^A^	38.4 ± 1.8^A^	43.8 ± 0.9^BC^
	16W + 8BFR	2361 ± 141^A^	28.8 ± 1.8^A^	40.7 ± 0.4^A^	45.3 ± 0.8^ABC^
	16W + 8FR	2430 ± 100^A^	30.7 ± 1.1^A^	41.6 ± 0.4^A^	41.8 ± 0.7^C^
Gina	16W	2567 ± 144^AB^	35.2 ± 2.5^A^	32.3 ± 1.0^B^	47.8 ± 1.5^A^
	24W	2953 ± 138^A^	31.8 ± 2.1^AB^	40.3 ± 1.0^A^	43.1 ± 0.4^AB^
	16W + 8B	2852 ± 117^A^	31.4 ± 1.6^AB^	41.2 ± 1.9^A^	41.1 ± 1.7^B^
	16W + 8BFR	2526 ± 136^AB^	27.3 ± 0.7^B^	39.5 ± 0.6^A^	43.2 ± 0.4^AB^
	16W + 8FR	2322 ± 88^B^	27.1 ± 1.2^B^	41.1 ± 0.8^A^	42.4 ± 0.7^B^
Eurix	16W	2568 ± 173^A^	35.0 ± 2.3^A^	32.4 ± 0.9^C^	44.0 ± 0.6^A^
	24W	2420 ± 132^A^	32.1 ± 1.1^AB^	38.3 ± 1.0^ABC^	44.0 ± 0.7^A^
	16W + 8B	2709 ± 126^A^	36.6 ± 2.7^A^	37.6 ± 2.5^BC^	41.7 ± 0.7^AB^
	16W + 8BFR	2451 ± 119^A^	28.0 ± 1.1^B^	44.0 ± 0.6^AB^	41.8 ± 0.8^AB^
	16W + 8FR	2574 ± 78^A^	31.7 ± 1.1^AB^	44.9 ± 1.0^A^	39.4 ± 0.7^B^
**64–66 DIT**					
Maureno	16W	4523 ± 63^C^	55.3 ± 1.6^A^	21.1 ± 0.6^D^	61.4 ± 1.2^A^
	24W	5535 ± 217^B^	51.8 ± 1.0^AB^	25.5 ± 0.7^C^	57.6 ± 1.4^AB^
	16W + 8B	6776 ± 254^A^	55.8 ± 1.6^A^	30.2 ± 0.8^AB^	55.4 ± 1.6^B^
	16W + 8BFR	6003 ± 96^B^	50.7 ± 1.0^AB^	28.9 ± 0.3^B^	59.4 ± 0.9^AB^
	16W + 8FR	6169 ± 117^AB^	49.7 ± 0.6^B^	31.7 ± 0.7^A^	54.9 ± 0.4^B^
Gina	16W	5229 ± 113^D^	52.0 ± 1.3^A^	23.2 ± 0.4^D^	61.7 ± 2.5^A^
	24W	5847 ± 298^CD^	52.5 ± 2.2^A^	27.1 ± 0.9^C^	56.1 ± 0.7^AB^
	16W + 8B	7399 ± 294^A^	51.2 ± 2.0^AB^	33.2 ± 0.8^AB^	51.1 ± 0.9^BC^
	16W + 8BFR	7079 ± 147^AB^	48.3 ± 0.9^B^	32.2 ± 0.9^B^	54.7 ± 0.7^BC^
	16W + 8FR	6433 ± 107^BC^	48.7 ± 1.0^B^	36.5 ± 0.4^A^	49.7 ± 0.7^C^
Eurix	16W	4908 ± 123^C^	58.5 ± 2.4^A^	21.1 ± 0.4^C^	53.4 ± 1.5^A^
	24W	5888 ± 196^B^	51.3 ± 1.8^AB^	26.5 ± 0.7^B^	47.9 ± 2.2^AB^
	16W + 8B	6826 ± 114^A^	54.8 ± 1.2^AB^	29.9 ± 0.6^A^	51.4 ± 1.1^AB^
	16W + 8BFR	5938 ± 164^B^	49.7 ± 1.0^B^	30.8 ± 0.5^A^	47.7 ± 1.0^AB^
	16W + 8FR	6888 ± 158^A^	54.8 ± 1.4^AB^	31.8 ± 0.3^A^	46.2 ± 0.7^B^

*Values and standard errors represent 10 plants during the 37–39 DIT measurement period and six plants during the 64–66 DIT measurement period. Within each parameter, cultivar, and measurement period, letter groups (A, B, C, D) represent a statistical difference as determined by a one-way ANOVA with a Tukey-Kramer adjustment (p < 0.05).*

At 37–39 DIT, the specific leaf area (SLA) of plants grown under four CL treatments in all three cultivars was higher than that of plants grown under 16W, indicating CL treatments reduced leaf thickness (Table 3). In ‘Maureno’ and ‘Gina,’ SLA was similar among all four CL treatments. In ‘Eurix,’ the plants under 16W + 8FR had higher SLA than that of plants under 16W + 8BFR (Table 3). At 64–66 DIT, a much clearer trend emerged where 16W + 8FR consistently produced the highest SLA while 16W produced the lowest SLA followed by 24W in all cultivars. At 37–39 DIT, the SPAD value, a metric closely correlated with chlorophyll content, was consistently the lowest in plants grown under 16W + 8FR and the highest in plants grown under 16W in all cultivars (Table 3). This trend continued during the 64–66 DIT measurements (Table 3). Taking all the information on leaf area, SLA, and SPAD into consideration, it seems that the plants grown in CL treatments adapted to the low light environment by reducing leaf thickness and increasing leaf area to improve light interception, especially for plants grown under 16W + 8FR.

### Regression Analysis of Biomass Partitioning and Internode Length to Phytochrome Photostationary State

**FIGURE 10:**
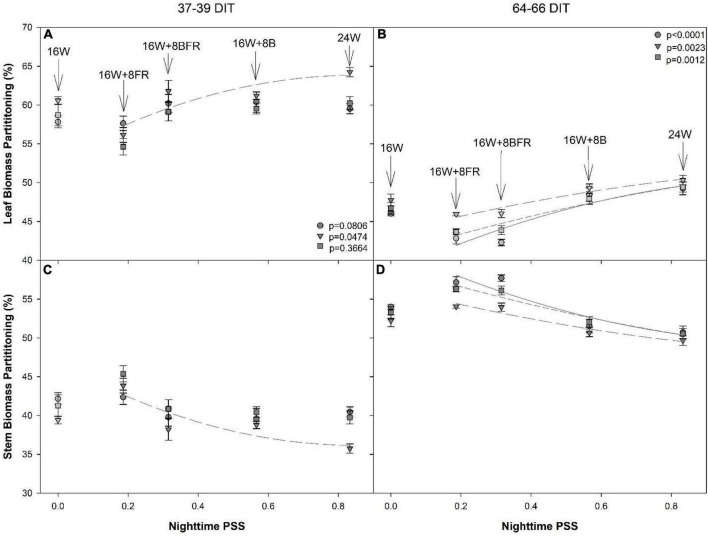
Regression analysis of biomass partition to leaf **(A,B)** and stem **(C,D)** in dry weight from ‘Maureno’ (circle; solid line), ‘Gina’ (downward triangle; long dash line), and ‘Eurix’ (square; short dash line) during the 37–39 DIT measurements **(A,C)** and 64–66 DIT measurements **(B,D)**. Nighttime PSS of 0.833, 0.566,0.315, and 0.186 correspond to treatments 24W, 16W + 8B, 16W + 8BFR, and 16W + 8FR respectively as determined *via* Sager et al. (1988). Regression analysis was done using a backward elimination method. The *p*-values listed are for the final regressions and only regressions which were determined to be significant (*p* < 0.05) are shown. The 16W treatment did not have light at night and thus was non-resultant during the PSS calculation, therefore, it was left out of the regression analysis. The biomass partition values for 16W are plotted in the graph at a PSS value of zero.

Regression analysis was conducted using the nighttime PSS values from CL treatments only where PSS was 0.833, 0.566, 0.315, and 0.186 for 24W, 16W + 8B, 16W + 8BFR, and 16W + 8FR, respectively. The 16W treatment was not included in the analysis due to the non-resultant quotient of the equation (i.e., because there was no light during the nighttime). A significant relationship between dry matter biomass partitioning to leaves and increasing PSS was only apparent for ‘Gina’ (Figure 10A). Conversely, the biomass partitioning to the stem tended to decrease with increasing PSS and was significant for ‘Gina’ only (Figure 10C). During the 64–66 DIT measurement period, a more obvious trend emerged as the dry biomass partitioning to the leaves increased with increasing PSS and biomass partitioning to the stem decreased with increasing PSS in all cultivars. Both fresh and dry biomass partitioning to leaves increased with increasing nighttime PSS and conversely partitioning to the stem decreased with increasing PSS (Figures 10B,D).

**FIGURE 11:**
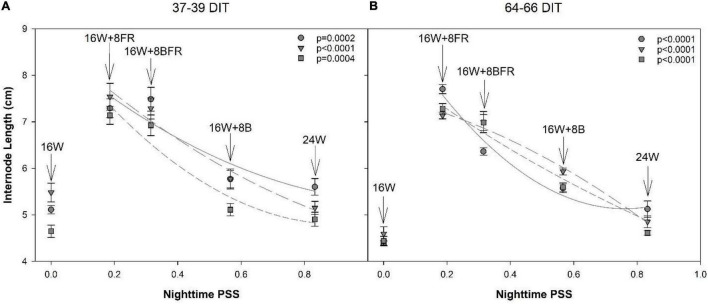
Regression analysis of internode length from ‘Maureno’ (circle; solid line), ‘Gina’ (downward triangle; long dash line), and ‘Eurix’ (square; short dash line) during the 37–39 DIT measurements **(A)** and 64–66 DIT measurements **(B)**. Nighttime PSS of 0.833, 0.566, 0.315, and 0.186 correspond to treatments 24W, 16W + 8B, 16W + 8BFR, and 16W + 8FR respectively as determined *via* Sager et al. (1988). Regression analysis was done using a backward elimination method. The *p*-values listed are for the final regressions and only regressions which were determined to be significant (*p* < 0.05) are shown. The 16W treatment did not have light at the night and thus was non-resultant during the PSS calculation. Therefore, it was left out of the regression analysis. The internode lengths for 16W are plotted in the graph at a PSS value of zero.

A decrease in PSS can enact a shade avoidance response characterized by increased stem elongation and leaf expansion. Here, the alternation of PSS occurred during the nighttime spectral shifts and also showed a shade avoidance response. As PSS decreased, the internode length of all cultivars at both measurement periods increased (Figure 11). Notably, below a PSS of 0.315 (16W + 8BFR), a further increase in internode length was not observed with the exception of the cultivar ‘Maureno’ during the 64-66 DIT measurement period (Figure 11B). Although the use of sole FR (16W + 8FR) further decreased the PSS compared to blue + FR (16W + 8BFR), a stronger shade avoidance response (in this case internode length) was not observed. This indicates that a PSS of 0.315 is sufficient to maximize stem elongation and no further increase in stem elongation was observed below this value. Taken together, an increased partitioning to the stem as the PSS decreases can be correlated with the increase in stem elongation under these treatments (Figures 9–11).

## Discussion

### Impact of Dynamic Continuous Lighting on Plant Injury and Yield

The implementation of CL during greenhouse crop production offers an intriguing option for growers. By utilizing light during the night period, the lower light intensity can be used during the daytime while still achieving the desired DLI target (Hao et al., 2018a). This translates to a reduced fixture requirement leading to vast capital cost savings for growers. However, CL means constant photon pressure on the plant which has been shown to cause photoperiod injury in both peppers and tomatoes leading to a reduction in fruit production (Hillman, 1956; Velez-Ramirez et al., 2014; Matsuda et al., 2016; Haque et al., 2017). Therefore, the feasibility of CL strategies hinges on whether the production (yield and quality) is equal to or greater than the conventional 16 h photoperiod lighting (control, 16W in this study). Due to their unique attributes, LEDs allow for the use of a dynamic CL strategy which employs a reduction in light intensity and/or change in spectral composition between day and night periods, which has been shown to eliminate or reduce injury in lettuce (Ohtake et al., 2018), tomato (Matsuda et al., 2016; Lanoue et al., 2019; Pham and Chun, 2020), and cucumber (Lanoue et al., 2021b). In this study with peppers, we showed that plants grown under two dynamic CL strategies (16W + 8B and 16W + 8BFR) were able to avert injury and maintain fruit yield and size (grade) when compared to the 16h control (16W; Figures 3, 5, 7). Both 16W + 8B and 16W + 8BFR employed a daytime PAR reduction of 18.2% while 16W + 8BFR also included a 25% reduction in daytime FR used; a total reduction of 18.9% in the extended PAR (ePAR; 400–780 nm) region (Table 1). In addition to the reduction in light fixture costs, electricity costs could also be reduced using the CL strategies because the electricity price is usually higher during the peak daytime than during the off-peak period at night in many regions in the world. Notably, both 16W + 8B and 16W + 8BFR which used dynamic CL performed better than 24W which maintained constant light intensity and spectrum during the 24h period (Figures 3G, 5). This indicates that the dynamic nature of the CL used in this study is essential for removing injury and sustaining fruit production in greenhouse pepper.

In contrast to our current study, the use of monochromatic blue light at 100 μmol m^–2^ s^–1^ (Velez-Ramirez et al., 2017) or 150 μmol m^–2^ s^–1^ (Matsuda et al., 2016) during the night produced a high degree of injury in tomatoes. However, our previous studies on tomato (Lanoue et al., 2019) and cucumber (Lanoue et al., 2021b) differ from this trend and indicate that the use of low intensity monochromatic blue light (50 μmol m^–2^ s^–1^) during the night was able to avert injury. In this study, nighttime blue light was considerably higher than the light compensation point (Table 2), and between that used in Velez-Ramirez et al. (2017) and Lanoue et al. (2019), and drove appreciable amounts of photosynthesis in 16W + 8B and 16W + 8BFR during the night (Figure 3B) without causing injury (Figures 4, 5). It should be noted that although 24W used a constant light intensity and spectrum that drove high rates of photosynthesis during the night and injury was observed, it was less than what was reported in other studies with other species (Hillman, 1956; Velez-Ramirez et al., 2014; Matsuda et al., 2016). This indicates that peppers may be less susceptible to long photoperiod injury than other species as previously noted by Demers and Gosselin (1999, 2002).

It is well known that light and temperature interaction has an effect on photosynthesis and yield; in general, an increase in light intensity requires an increase in temperature to maintain an optimum homeostatic balance and drive assimilation (Walters and Lopez, 2021; Song et al., 2022). In our study, nighttime temperatures were not optimized for all four CL treatments, specifically the 24W which had the highest nighttime light intensity. This was because there is a lack of information on optimum temperature management for dynamic CL treatments, and all light treatments were in the same greenhouse and temperature could not be individually adjusted within each treatment. However, the greater SLA in all four CL treatments than 16W (control; Table 3) is an indication of the air temperature may be too high for the CL treatments. The plant temperature, especially at the top canopy, might be higher for plants grown under CL because of the continuous exposure to light, when at the same air temperature. Therefore, the optimum air temperature for CL treatments may be lower. Haque et al. (2015, 2017) observed that tomato plants grown under CL with a reduced nighttime temperature (16°C vs. 23°C in the control) had little to no leaf injury under continuous illumination. Hao et al. (2018b,2020) discovered the response of greenhouse tomatoes, peppers, and cucumbers to long photoperiods of HPS lighting is improved by a temperature drop during the first 3 h after the lighting is off and the temperature control strategy reduced heating energy demand. Leaf injury in tomatoes and peppers is reduced by the temperature drop (Hao et al., 2018b). Therefore, further research is needed to investigate the interaction between temperature control and dynamic CL to determine the optimum air temperature and temperature control strategy for dynamic CL lighting.

### The Impact of Nighttime Spectra on Plant Growth

Although the DLI was not controlled for, Demers et al. (1998) observed a significant reduction in plant height, internode length, and leaf area in peppers grown under supplemental HPS light for 24 h compared to 14 h. Velez-Ramirez et al. (2014) also noted that even if tomatoes were genetically altered to eliminate the occurrence of CL injury, a reduction in leaf area would hinder plant performance in a practical setting due to reduced light capture. However, the use of sole blue light, such as the application of blue light during the night, has been shown to elicit a response similar to the shade avoidance response of FR (Hernández and Kubota, 2016; Lanoue et al., 2019; Kong and Zheng, 2020). In experiment two, plant height was seen to be greater when plants were grown under 16W + 8B which contained sole blue light during the night period compared to the 16 h control (Figure 9). It was also noted that this phenomenon was time-dependent as the difference in plant height increased after the treatments were applied for a longer time (Figure 9B). This confirms the studies in tomato and *Arabidopsis* which indicate sole blue light is able to increase plant height (Hernández and Kubota, 2016; Lanoue et al., 2019; Kong and Zheng, 2020). The increase in plant height coupled with an increase in leaf area and specific leaf area indicate that sole illumination with blue light during the night can induce a shade avoidance response (Table 3). Not only does this have an impact on plant architecture, but can also allow the plant to better capture light during the daytime period (Hersch et al., 2014).

Far-red light has long been known to elicit a strong photomorphogenic response in plants (Franklin and Whitelam, 2005). Here, we observed that the use of sole FR during the night period resulted in the largest stem elongation of all treatments in peppers (Figure 9). Interestingly, it was determined that the movement of just 25% of the total FR DLI from the daytime (16W) to the nighttime (16W + 8BFR and 16W + 8FR) at only 10–11 μmol m^–2^ s^–1^ was able to produce a significantly stronger photomorphogenic response than the use of FR during the daytime only. Indeed, this time-specific use of FR light led to an increase in internode length of 37.6–53.5% during the first destructive measurement and 55.6–75.8% during the second destructive measurement, depending on cultivars. All treatments within experiment two utilized a similar total FR DLI of approximately 1.09 mol m^–2^ d^–1^ (Table 1).

During the initial destructive measurements, dry matter partitioning to the leaves was observed to increase with decreasing PSS only in the ‘Gina’ cultivar. However, during the subsequent destructive measurements, all plants showed increasing biomass partitioning to the leaves when PSS was decreased. This result is opposite to what was observed in lettuce plants grown with or with sole-source FR (Zou et al., 2021). Species-specific responses to FR could arise due to the differences in plant architecture. Whereas lettuce is a relatively small plant that mainly grows low to the ground and has little internode length, peppers are a vine-type crop grown vertically during production. Therefore, it is likely that FR light is able to penetrate deeper into the pepper crop causing the effect. Further to this, the peppers in our study were under the different treatments for more than 2 months, much longer than the lettuce in Zou et al. (2021) and thus, a time-dependent FR response was also observed which may account for the differences observed.

In this study, there was a significant reduction in PSS during the nighttime from 24W (PSS = 0.833) to 16W + 8FR (PSS = 0.135) allowing for the evaluation of the impact of nighttime FR and PSS on internode length and stem elongation. It was determined that the time-of-use, in this case with or without PAR (daytime or nighttime), can drastically impact the strength of photomorphogenic response as also noted in lettuce (Zou et al., 2021). The use of nighttime FR can have a much larger impact on plant morphology than utilization with broad-spectrum light (i.e., during the daytime) as internode length was seen to increase with decreasing PSS (Figure 11). A similar internode lengthening was also observed when PSS was decreased in lisianthus when grown using a 5-h night interruption (Yamada et al., 2011), although the study used fluence rates of only 3 μmol m^–2^ s^–1^. The plants in our study had a stronger morphological response to the use of nighttime FR than has been previously reported with the use of EOD-FR (Kalaitzoglou et al., 2019). In Kalaitzoglou et al. (2019), the PSS of the EOD-FR was 0.1 which was similar to our lowest nighttime PSS of 0.135 (16W + 8FR) but much higher than the PSS of 0.315 (16W + 8BFR). However, we saw a much more dramatic increase in internode length than previously reported (Kalaitzoglou et al., 2019) and also at a higher PSS (0.315). This indicates that the response to FR light is also dependent on the length of use and not simply a dose-response. It should also be noted that Kalaitzoglou et al. (2019) used tomato as their model whereas our study looked at pepper. This, along with the results from Zou et al. (2019, 2021) indicates that species, and potentially even cultivars within a species, react differently to nighttime FR light. Furthermore, the increase in plant height also corresponded to an increase in biomass partitioning to the stem (Figures 10C,D) which is supportive of previous works (Brown et al., 1995; Ji et al., 2019, 2020).

It is also noteworthy that similar to 16W + 8BFR, the 24W treatment included a movement of 25% of FR from the day to the night (Table 1). However, an increase in plant height was not observed when compared to 16W with the exception of ‘Maureno’ during the 64–66 DIT measurements (Figure 8B). The 24W and 16W + 8BFR treatments contained the same amount of FR light during the night, however, an increase in stem elongation was only observed in the latter. The notable difference between the two nighttime light treatments is the PAR intensity and spectrum, as well as PSS. The 24W treatment has approximately doubled the PAR intensity compared to 16W + 8BFR, has a broad-spectrum light as opposed to only blue light, and has a drastically higher PSS value. A lower red:far-red, correlated with a lower PSS, will invoke a shade avoidance response resulting in the greater stem elongation and leaf expansion observed in 16W + 8BFR compared to 24W (Franklin and Whitelam, 2005). Furthermore, the response to FR is dampened even if PSS is controlled for when used in a higher light intensity environment (Kalaitzoglou et al., 2019). These two factors are likely responsible for the different responses to FR during the night between 24W and 16W + 8BFR. However, the lack of further increase in internode length from 16W + 8BFR (PSS = 0.315) to 16W + 8FR (PSS = 0.135) indicates that there may be a threshold (PSS = 0.315) at which a further reduction in PSS does not increase internode length. A PSS value of 0.315 is comparable to the PSS threshold (PSS = 0.23) determined for arugula and mustard microgreens beyond which no further stem elongation was observed (Ying et al., 2020). Therefore, with respect to plant height and internode length, FR during the night had a larger impact on the morphological outcome as plants subjected to 16W + 8BFR tended to have values more closely to 16W + 8FR (Figure 9). On the other hand, plants under 16W + 8BFR had a total leaf area closer to that of 16W + 8B plants indicating blue at night supported leaf expansion (Table 3). This indicates that the two shade avoidance processes may be controlled by different mechanisms which can be preferentially controlled by blue or FR independently.

### Implication on Greenhouse Pepper Production

Short internode lengths during lit pepper production have been well documented and can have negative implications during plant maintenance and fruit harvest. The use of extended (8 h) periods of FR light during the night resulted in an improved canopy architecture with increased internode length, preventing fruit stacking, and could reduce labor costs for plant maintenance (Figure 11). The addition of FR in 16W + 8BFR and 16W + 8FR during the night created a more “open” canopy. This allows workers a better view of the plant material allowing for quicker and more precise determination of offshoot and suckers and faster removal of offshoot/sucker by hand, reducing the labor time for routine maintenance. The stem elongation observed with the use of nighttime FR also creates a larger distance between nodes. During fruit growth, this provides more room for the fruit to grow unimpeded. As opposed to the 2.9 cm internodes observed by Demers et al. (1998), the use of FR at night extended internode length close to 8 cm providing ample room for normal pepper fruit growth.

Light pollution from greenhouses has been seen as a nuisance to neighboring municipalities including residents and businesses. Recently, bylaws have been enacted in Ontario, Canada, and Netherlands mandating the use of light abatement curtains to prevent light emissions from the greenhouses during the night (Hanifin, 2019). Light abatement curtains not only block light but also block 50–70% of heat loss from a greenhouse (Svensson, 2020), leading to overheating and high-temperature stress to crops due to the buildup of heat from lighting application, especially in greenhouse fruit vegetable production. For greenhouse tomato, pepper, and cucumber production, conventional lighting strategies usually use about 200–250 μmol m^–2^ s^–1^ light for 16 h (such as 1:00 to 17:00 or 0:00 to 16:00, Hao et al., 2018a). To prevent crop damage caused by high temperature, the curtains need to be partially opened (so-called “gapping”) to ventilate out the heat when the outside temperature is not cold enough to bring down the greenhouse temperature. This is not feasible with the bylaw in Netherlands. The bylaw mandates the blocking of 98% of light during the night if the light intensity is > 15,000 lux (about 183 μmol m^–2^ s^–1^ for HPS, Ashdown, 2020). The use of low intensity of lighting in together with LEDs can eliminate this issue, allowing the curtains to be fully closed to prevent light emission from the greenhouse during the night.

It has been demonstrated that dynamic CL (i.e., 16W + 8B and 16W + 8BFR) did not harm the plants and led to a similar yield when compared to the control (16W). Notably, 16W + 8BFR did produce a much more open canopy which has positive labor benefits as mentioned above. While smart LEDs with the capability to control both spectrum and intensity are generally more complex leading to higher initial cost per light fixture than the LED fixture with fixed intensity and spectral composition at present time, dynamic low-intensity CL can help to reduce initial capital costs. In this study, 16W + 8BFR can reduce the intensity or installed capacity of light fixtures by 19% (Table 1). A dynamic CL using a smart 24 h LED system can reduce light intensity/fixture capacity by a third and is more cost-effective than a conventional 16 h LED system in mini-cucumber production based on the cost of electricity per unit of produce, in Ontario (Lanoue et al., 2021b). The benefit of the reduction in light intensity/fixture requirement with the dynamic low-intensity CL will not change regardless of the electrical prices during the day/night. Other studies that used fixed light spectral composition and dynamic light intensity control in response to sunlight intensity fluctuations have shown that electricity consumption can be reduced by 10–30% in comparison to a traditional on-off regime (Pinho et al., 2013; van Iersel and Gianino, 2017). The smart LED fixtures used for the implementation of dynamic CL in this study can control both light intensity and spectral compositions. Therefore, there is a good potential in future research and development to combine the sunlight-based intensity control strategy with the dynamic CL strategy for reducing both light fixture cost and electricity consumption.

## Conclusion

In conclusion, experiment 1 provided data that indicates that pepper plants can be grown under CL lighting without the leaf injury associated with a continuous photoperiod. Moreover, it was determined that in order to have injury-free production, a dynamic CL lighting strategy was needed. The application of either blue and/or FR light at night reduced PSS which led to a shade avoidance response and increase in internode length, and the response to the application of far-red during the night was much stronger than the application during the daytime. Moreover, when blue and FR were combined at night, the response was not additive but similar to 16W + 8FR. This indicates a potential threshold of PSS (0.315) beyond which no further stem elongation is enabled. In 16W + 8BFR, the use of blue light was able to drive photosynthesis during the night and blue + FR at night was able to evoke positive morphological responses reducing fruit stacking. Taken together, 16W + 8BFR, a treatment that provided white light during the day followed by both blue and FR during the night, is potentially the best CL for pepper production in this study, because it has the largest potential to reduce capital fixture cost, and daytime light intensity and electricity cost. Furthermore, its yield and fruit grade/quality were similar to 16W while also addressing the short internode issue in pepper production with supplemental lighting. While 16W + 8BFR did not improve the yield compared to 16W, it improved the canopy architecture making routine plant maintenance easier and faster, potentially reducing labor costs for producers.

## Data Availability Statement

The original contributions presented in the study are included in the article/Supplementary Material, further inquiries can be directed to the corresponding author/s.

## Author Contributions

JL and XH were involved in the conceptualization, methodology development, and writing and editing of the manuscript. JL and CL were involved in data curation and the day-to-day upkeep of the experiment. JL performed the data analysis. XH was responsible for funding acquisition. All authors have read and agreed to the submitted manuscript.

## Conflict of Interest

The authors declare that the research was conducted in the absence of any commercial or financial relationships that could be construed as a potential conflict of interest.

## Publisher’s Note

All claims expressed in this article are solely those of the authors and do not necessarily represent those of their affiliated organizations, or those of the publisher, the editors and the reviewers. Any product that may be evaluated in this article, or claim that may be made by its manufacturer, is not guaranteed or endorsed by the publisher.

## Footnotes

1
https://en.wikipedia.org/wiki/Photosynthetic_efficiency

2
https://energyeducation.ca/encyclopedia/Law_of_conservation_of_energy
