# Supplementary material for: The Power of Far-Red Light at Night: Photomorphogenic, Physiological, and Yield Response in Pepper During Dynamic 24 Hour Lighting
Source: Front Plant Sci. 2022 Apr 26;13:857616. doi: 10.3389/fpls.2022.857616 (PMC9087831; doi:10.3389/fpls.2022.857616)
Supplement: Supplementary file 1 [file Data_Sheet_1.PDF]

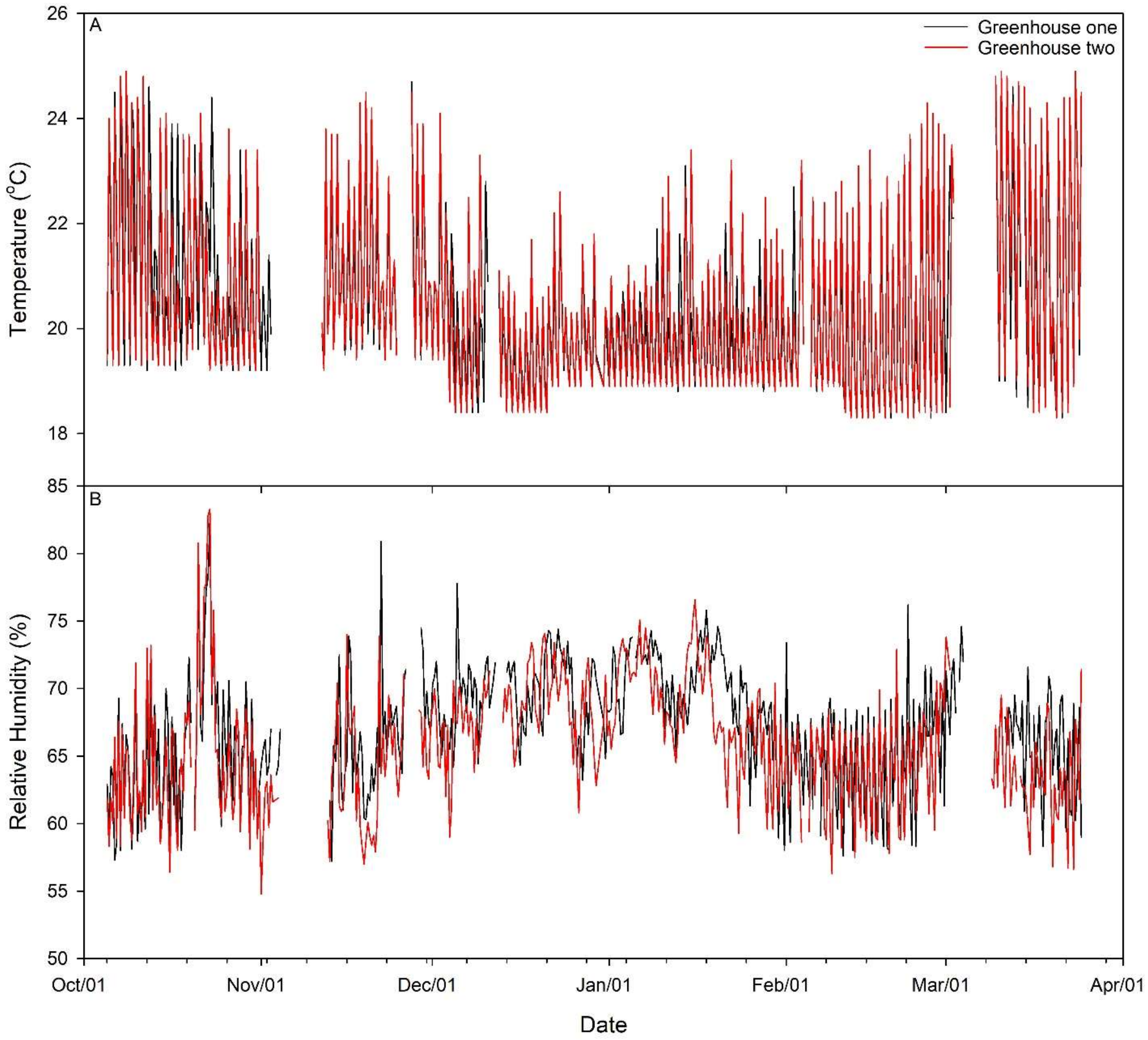

Supplementary Figure 1: Temperature (A) and relative humidity (B) inside both greenhouses during experiment one (October 5th, 2020 – March 24th, 2021).

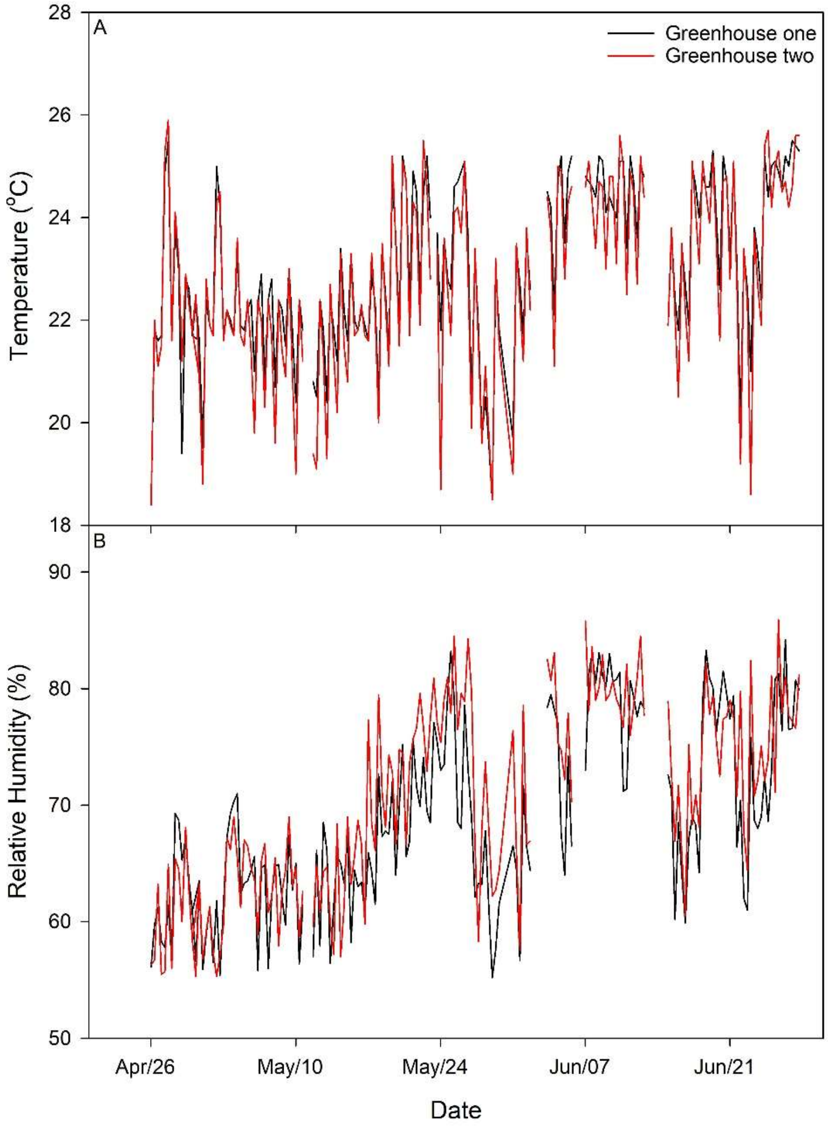

Supplementary Figure 2: Temperature (A) and relative humidity (B) inside both greenhouses during experiment two (April 26th, 2021 – June 30th, 2021).
